# Effective Distance between Aortic Valve and Conduction System Is an Independent Predictor of Persistent Left Bundle Branch Block during Transcatheter Aortic Valve Implantation

**DOI:** 10.3390/medicina57050476

**Published:** 2021-05-11

**Authors:** Thomas T. Poels, Robert Stassen, Suzanne Kats, Leo Veenstra, Vincent van Ommen, Bastiaan Kietselaer, Patrick Houthuizen, Jos G. Maessen, Frits W. Prinzen

**Affiliations:** 1 Department of Cardiothoracic Surgery, Maastricht University Medical Center, P.O. Box 5800, 6229HX Maastricht, The Netherlands; r.c.stassen@gmail.com (R.S.); suzanne.kats@mumc.nl (S.K.); j.g.maessen@mumc.nl (J.G.M.); 2 Department of Cardiology, Maastricht University Medical Center, P.O. Box 5800, 6229HX Maastricht, The Netherlands; l.veenstra@mumc.nl (L.V.); v.van.ommen@mumc.nl (V.v.O.); b.kietselaer@mumc.nl (B.K.); 3 Department of Cardiology, Catharina Hospital Eindhoven, P.O. Box 1350, 5623EJ Eindhoven, The Netherlands; patrick.houthuizen@catharinaziekenhuis.nl; 4 CARIM School for Cardiovascular Diseases, P.O. Box 616, 6229ER Maastricht, The Netherlands; frits.prinzen@maastrichtuniversity.nl

**Keywords:** transcatheter aortic valve implantation, left bundle branch block, predictor, computed tomography scan

## Abstract

*Background and objectives:* Persistent left bundle branch block (P-LBBB) has been associated with poor clinical outcomes of transcatheter aortic valve implantation (TAVI) procedures. We hypothesized that the distance from the aortic valve to the proximal conduction system, expressed as the effective distance between the aortic valve and conduction system (EDACS), can predict the occurrence of P-LBBB in patients undergoing a TAVI procedure. *Materials and methods:* In a retrospective study, data from 269 patients were analyzed. EDACS was determined using two longitudinal CT sections. *Results:* Sixty-four of the patients developed P-LBBB. EDACS ranged between −3 and +18 mm. EDACS was significantly smaller in P-LBBB than in non-P-LBBB patients (4.6 (2.2–7.1) vs. 8.0 (5.8–10.2) mm, median values (interquartile range); *p* < 0.05). Receiver operating characteristic analysis showed an area under the curve of 0.78 for predicting P-LBBB based on EDACS. In patients with EDACS of ≤3 mm and >10 mm, the chance of developing P-LBBB was ≥50% and <10%, respectively. *Conclusions:* A small EDACS increases the risk for the development of P-LBBB during TAVI by a factor of >25. As EDACS can be measured pre-procedurally, it may be a valuable additional factor to weigh the risks of transcatheter and surgical aortic valve replacement.

## 1. Introduction

Transcatheter aortic valve implantation (TAVI) is a proven treatment option for patients with severe aortic valve stenosis [1,2]. An important complication of TAVI, however, is the creation of conduction abnormalities such as left bundle branch block (LBBB). LBBB develops in 7–65% of cases and persists in a third of cases [3,4,5,6,7,8]. As recently reviewed, there is strong evidence that TAVI-induced LBBB reduces the hemodynamic benefit of TAVI and increases the mortality rate, presumably due to dyssynchrony-induced heart failure and development of higher-degree atrioventricular (AV) block [9,10,11,12,13,14].

TAVI-induced LBBB is considered to develop due to damage of the conduction system created by the new valve prosthesis or by maneuvers during the valve implantation [6,9,10,14]. In post-procedural analyses, procedural factors such as the implantation depth (ID), a low ratio of annulus/balloon or annulus/prosthesis and self-expandable aortic valve prostheses have been shown to be important luxating factors [9,13,14]. Patient-related factors that increase the risk for developing LBBB include aortic valve calcifications, increased QRS duration, pre-existent right bundle branch block (RBBB), diabetes and peripheral vascular disease [13].

**Figure 1:**
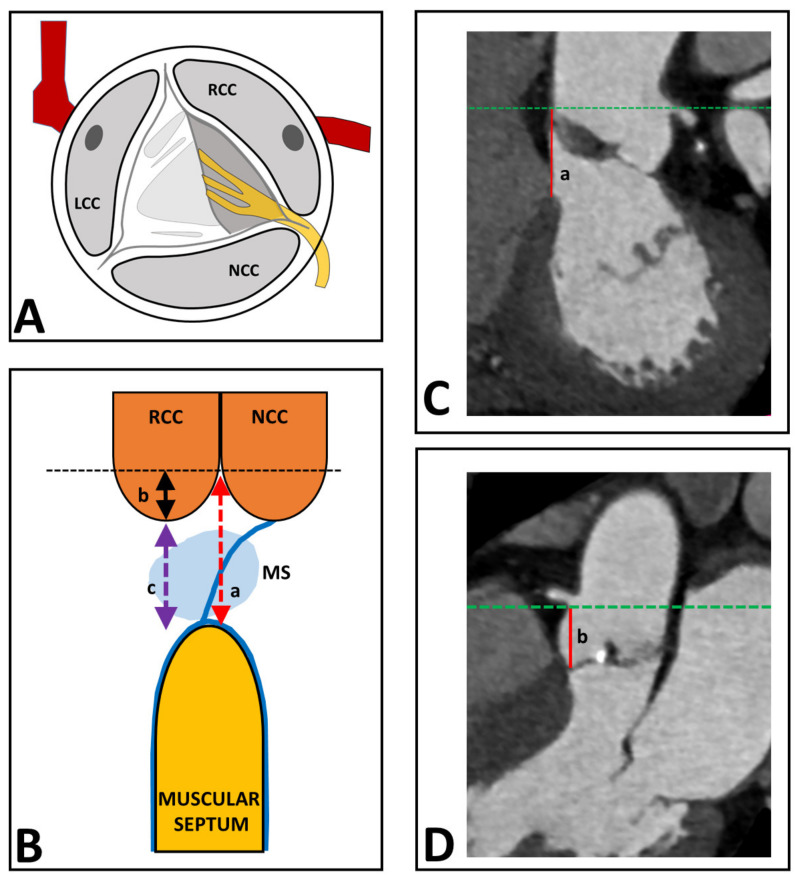
CT analysis of determination of EDACS from the CT scan. (**A**) shows the His-bundle that runs from the aortic valve (between the NCC and RCC) in an oblique trajectory towards the muscular septum. (**B**) shows the distance between the sinotubular junction and the muscular septum (line a), the depth of the RCC, measured from the sinotubular junction to the deepest point of the RCC (line b), and EDACS, which is calculated as the difference between line a and b (line c). (**C**) shows the distance between the sinotubular junction and the muscular septum (line a) measured on the CT scan. (**D**) shows the depth of the RCC, measured from the sinotubular junction to the deepest point of the RCC (line b) on the CT scan. EDACS, effective distance between aortic valve and conduction system; LCC, left coronary cusp; MS, membranous septum, NCC, non-coronary cusp; RCC, right coronary cusp.

It was the aim of the present study to investigate to what extent the distance between the aortic valve and the proximal conduction system may predict the development of LBBB during the TAVI procedure. The His-bundle is intimately related to the base of the interleaflet triangle between the non-coronary (NCC) and right coronary (RCC) cusps of the aortic valve, while more distally being more intimately related to the right coronary aortic cusp (Figure 1A). It splits into a right and a left bundle branch near the crest of the muscular septum [15,16,17]. Therefore, a smaller distance between the aortic valve and the muscular septum may make patients more vulnerable to the occurrence of conduction abnormalities during a TAVI procedure. This idea is supported by previous studies, showing that a shorter membranous septum length is predictive of permanent pacemaker implantation [18,19].

It was the goal of the present study to develop a robust measure of the effective distance between the aortic valve and conduction system (EDACS) on the preoperative CT scan and to investigate whether this EDACS is a patient-specific anatomical predictor for the occurrence and persistency of LBBB during a TAVI procedure. In this analysis, we also accounted for valve type (balloon-expandable (i.e., Edwards-Sapien, Irvine, CA, USA) and self-expandable valves (i.e., Medtronic CoreValve, Minneapolis, Minnesota, USA)), valve size and implantation depth.

## 2. Materials and Methods

### 2.1. Study Design and Population

This study was performed at the Maastricht University Medical Center and at the Catharina Hospital Eindhoven. All patients who underwent a TAVI procedure between January 2011 and June 2016 were included. This time window of inclusion was chosen as in 2011, acquisition of cardiac computed tomography (CT) scans for preoperative planning was started and the end date of June 2016 provided us with a minimum follow-up of half a year in all patients. Exclusion criteria were a pre-existing permanent pacemaker (PPM), a wide QRS complex at baseline and prior implantation of an aortic valve prosthesis.

Electrocardiographic data recorded preoperatively, postoperatively, at discharge and at follow-up at 6–12 months were prospectively collected in a central registry. The medical ethics committee of the hospital waived the need for informed consent.

This retrospective study used data that are routinely acquired for standard healthcare purposes. Therefore, the ethical committee confirmed that the Medical Research Involving Human Subjects Act (WMO) does not apply to this study and no informed consent was required. Patients who had signed a general objection to use their medical data were not included in the study.

### 2.2. ECG

All ECGs were reviewed for LBBB. According to the established Strauss criteria, LBBB was defined as a QRS duration of >140 ms (men) or >130 ms (women), QS or rS in leads V1 and V2 and mid-QRS notching or slurring in 2 of leads V1, V2, V5, V6, I and aVL [20].

Any new LBBB which was present postoperatively but disappeared at discharge or follow-up was defined as transient (T). If the BBB was still present on the follow-up ECG, it was considered persistent (P).

### 2.3. CT Data Analysis for EDACS

For each patient, a retrospective gated multiple-detector CT angiography from the heart and aortic root was obtained for preoperative planning of the TAVI procedure. Images were analyzed using a multi-plane reconstruction viewer. The systolic phases between 10% and 30% of the cardiac cycle were chosen as these showed the best reproducibility.

The conduction system cannot be visualized on the CT directly; therefore, we designed a derivative according to known anatomy. The His-bundle runs from the aortic valve (between the NCC and RCC) down the membranous septum towards the muscular septum (Figure 1A). Therefore, we used the EDACS as the surrogate for distance between the aortic valve and bundle branches. In the CT images, the aortic annulus was aligned in the coronal and sagittal view to the plane of the sinotubular junction (Figure 1). The distance between the sinotubular junction and the muscular septum was measured (line a in Figure 1B,C). Then, the coronal view was aligned to an oblique coronal view until the deepest point of the RCC was visible. The depth of the RCC was measured from the sinotubular junction to the deepest point of the RCC (line b in Figure 1B,D). EDACS was calculated as the difference between line a and b (line c in Figure 1B).

Implantation depth was measured using a Dicom-viewer (MicroDicom 2.2.5, Sofia, Bulgaria) to evaluate the angiography images from the implantation procedure. Depth of implantation was measured from the implanter’s view, and implantation depth was defined as the distance from the deepest point in the right coronary cusp to the ventricular edge of the frame.

As an LBBB may originate from damage by a prosthesis to the LBB, we calculated the difference between the EDACS and the implantation depth. This “EDACS-ID” was considered as a measure of how close the lower part of the prosthesis would be to the top of the ventricular septum in the individual patient.

### 2.4. Prostheses Types

In procedural factors, the TAVI prostheses were divided into “balloon-expandable” and “self-expandable” profile prostheses, with balloon-expandable prostheses being Edwards Sapien XT and −3 valves and the self-expandable prostheses containing the following: Medtronic CoreValve, Engager and Evolut-R as well as St. Jude Medical Portico and Symetis Acurate.

### 2.5. Statistical Analysis

Categorical variables are presented as numbers and proportions, and binary logistic regression analysis was used to compare categorical variables with two categories and the χ2 test for categorical data with more than two categories.

For continuous variables, normality of distribution was assessed with the Kolmogorov–Smirnov test. Normal and skewed continuous variables are presented as means with standard deviation and medians with interquartile range (IQR) and were compared using the Kruskal–Wallis test. A Bonferroni correction was applied for multiple comparisons.

A binary logistic regression analysis test was used to analyze predictors for the occurrence of TAVI-induced LBBB. Characteristics in both univariate analyses with a *p*-value less than 0.10 were included in the multivariate analyses.

All statistical analyses were performed using Statistical Package for Social Sciences, version 23 (IBM SPSS, Chicago, IL, USA). *p* < 0.05 was considered significant.

## 3. Results

**Figure 2:**
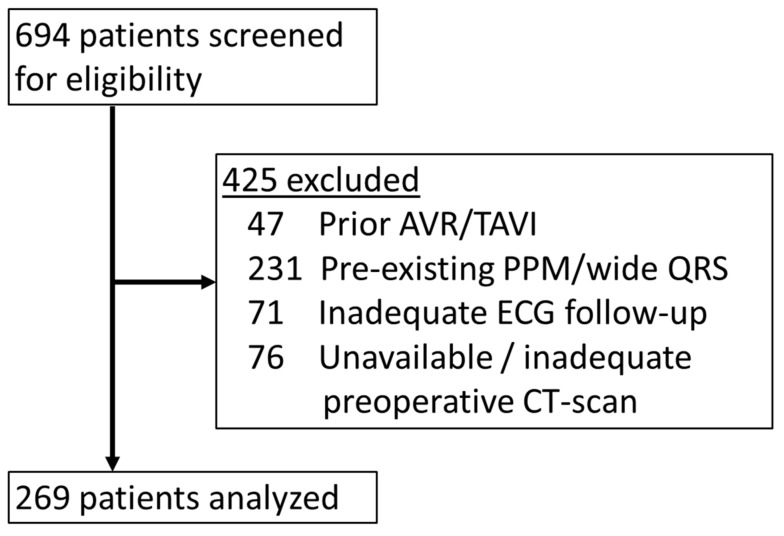
Inclusion of patients. Selection of the study population. PPM, permanent pacemaker; AVR, aortic valve replacement; TAVI, transcatheter aortic valve implantation; ECG, electrocardiogram; CT-scan, computed tomography scan.

After screening 694 patients, 269 patients were eligible for analysis (Figure 2). Table 1 shows the baseline characteristics of the patient cohort, consisting of mainly octogenarians, of whom 75.1% received a balloon-expandable valve. ECG analysis at follow-up showed that the cohort consisted of 64 P-LBBB and 205 non-P-LBBB patients (Table 1). Baseline characteristics only showed a small but significant difference in QRS duration between the groups (QRS P-LBBB 98(89–104) ms (median (IQR)) and QRS non-P-LBBB 94 (86–102) ms (median (IQR))). Procedural factors did not significantly differ between groups, except for prosthesis size (Table 1).

Of the 200 patients who received a balloon-expandable valve, 23% acquired a P-LBBB, while of the 69 patients receiving a self-expandable valve, 26% developed a P-LBBB.

The intra-observer variability of the EDACS measurement was small, with a mean value (SD) of 0.6 (1.7) mm and a correlation coefficient of 0.87, while inter-observer variability was 0.5 (2.2) mm (determined using 20 cases), indicating that EDACS can be assessed reproducibly.

### 3.1. The Effect of EDACS and Implantation Depth

In an initial analysis, patients were divided into narrow QRS (nQRS), T-LBBB and P-LBBB. EDACS was significantly smaller in patients who developed a P-LBBB than in patients with T-LBBB and nQRS (*p* < 0.05, Table 2), while EDACS was not significantly different between T-LBBB and nQRS patients. Additionally, implantation depth (ID) and the difference between EDACS and ID were not significantly different between T-LBBB and nQRS patients. Therefore, for the remainder of the analyses, data from T-LBBB and nQRS were pooled and are further referred to as non-P-LBBB.

**Figure 3:**
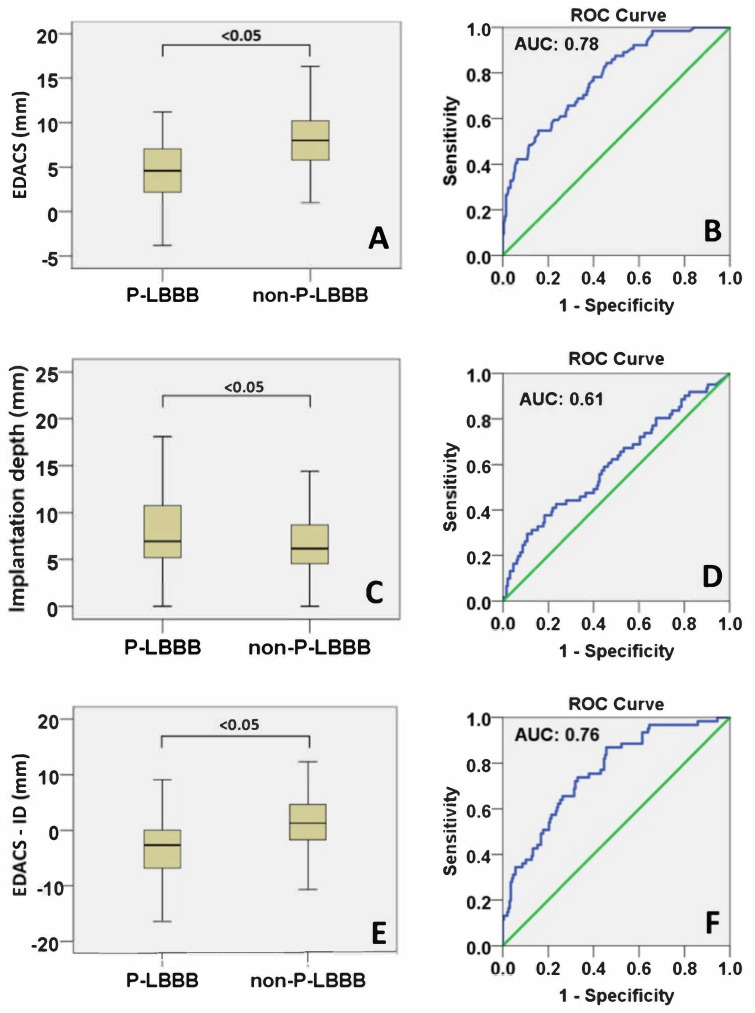
EDACS analyses for entire study population. Boxplots of effective distance between aortic valve and conduction system (EDACS) (**A**), implantation depth (**C**) and the difference between EDACS and implantation depth (EDACS-ID) (**E**) for the persistent left bundle branch block (P-LBBB) and non-P-LBBB patient groups. Corresponding to these boxplots are ROC curves for EDACS (**B**), implantation depth (**D**) and EDACS-ID as predictors of P-LBBB (**F**).

Within this patient cohort, EDACS ranged between −3 and +18 mm, the negative value indicating cases where the membranous septum (MS) started above the deepest point of the RCC (segment B being larger than segment A, Figure 1). Figure 3A shows that EDACS was significantly smaller in patients who developed P-LBBB than in those who did not (4.6 (2.2–7.1) vs. 8.0 (5.8–10.2), median (IQR), *p* < 0.05). ROC analysis showed an area under the curve (AUC) of 0.78 for predicting P-LBBB based on EDACS (Figure 3B).

**Figure 4:**
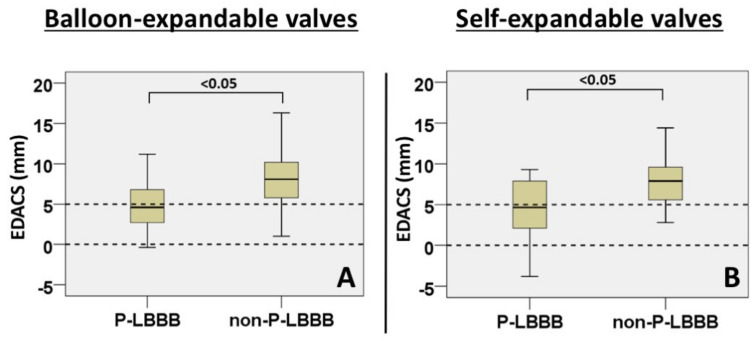
EDACS analyses between prosthesis type. Boxplots of EDACS for balloon-expandable valves (**A**) and self-expandable valves (**B**). P-LBBB, persistent left bundle branch block; non-P-LBBB, no persistent left bundle branch block. EDACS; effective distance between aortic valve and conduction system.

Implantation depth was significantly larger in P-LBBB than in non-P-LBBB patients (Table 3 and Figure 3C), and the AUC for developing P-LBBB was 0.61 (Figure 3D). However, using the difference between EDACS and implantation depth (EDACS-ID) did not increase the AUC as compared to that by EDACS alone (Figure 3E,F). EDACS was significantly different between P-LBBB and non-P-LBBB patients for both self-expandable and balloon-expandable valves (Figure 4A,B).

In univariate analysis, the odds ratios (ORs) of EDACS, prosthesis size and implantation depth with P-LBBB were significant (Table 4). In multivariate analysis, only EDACS remained significant (OR 0.68, 95% CI 0.60–0.77, *p* < 0.05) (Table 4).

### 3.2. Cut-Off Values for EDACS

**Figure 5:**
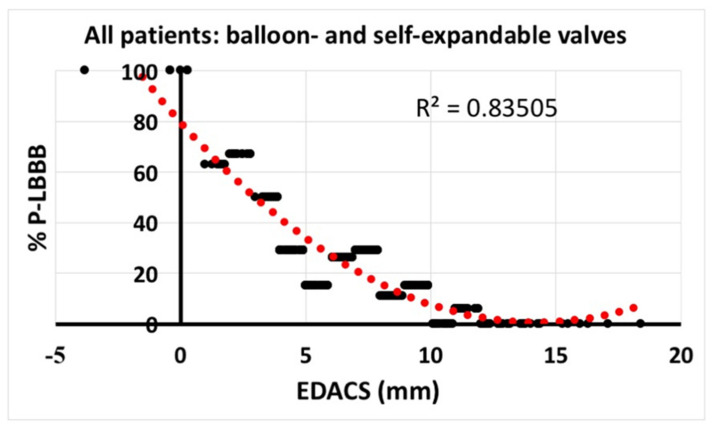
Correlation of EDACS and P-LBBB%. Relation between effective distance between aortic valve and conduction system (EDACS) and % persistent left bundle branch block (P-LBBB). To this purpose, all data were divided in bins of 1 mm of EDACS, where the more patients per bin, the wider the visual data points. mm, millimeter.

Grouping all patient data in bins for each millimeter of EDACS showed that an EDACS of ≤3 mm provided a >50% chance of developing P-LBBB (Figure 5). Conversely, an EDACS of ≥10 mm provided a chance of less than 10% of developing a P-LBBB. The positive (PPV) and negative predictive values (NPV) of EDACS of ≤3 mm for developing P-LBBB were 73.1% and 81.5%. The PPV of EDACS of ≥10 mm for avoiding P-LBBB was 98.1% and the NPV was 70.4%.

**Figure 6:**
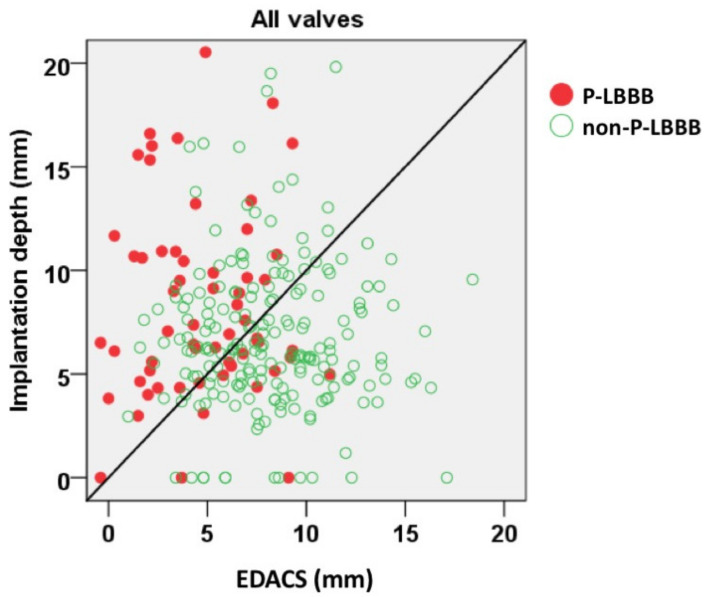
Implantation depth and EDACS. Correlation between implantation depth and effective distance between aortic valve and conduction system (EDACS) for all patients. Red symbols depict patients with P-LBBB; open symbols those without P-LBBB. Diagonal line is line of unity. EDACS, effective distance between aortic valve and conduction system; mm, millimeter; P-LBBB, persistent left bundle branch block; non-P-LBBB, no persistent left bundle branch block.

Figure 6 shows all individual data points for the relation between EDACS and implantation depth. Data points above the line of unity depict patients in whom the implantation depth was larger than the EDACS. Indeed, most of the P-LBBB patients were in this segment. The figure also depicts the high percentage of P-LBBB in patients with EDACS of ≤3 mm. Theoretically, a P-LBBB might be avoided by an implantation depth smaller than EDACS, but Figure 6 also shows that there were no patients with EDACS of ≤3 mm with an even smaller implantation depth.

## 4. Discussion

The main finding of this study is that there is a considerable variation in EDACS between patients and that a short EDACS is a positive predictor of development of P-LBBB during a TAVI procedure. An EDACS of ≤3 mm is associated with a ≥50% chance of occurrence of a P-LBBB, whereas patients with EDACS of >10 mm have hardly any risk for P-LBBB with the current practice. Prosthesis size and implantation depth significantly predict LBBB in univariate analysis, but not in multivariate analysis.

### 4.1. EDACS as a Predictor for P-LBBB

The finding that EDACS is an important predictor for the occurrence of P-LBBB fits with the idea that such conduction abnormalities originate from damage of the conduction system by the newly implanted prosthesis or from manipulations such as balloon inflations. After all, the smaller the EDACS, the closer the conduction system is to the native aortic valve and, therefore, to the site of the implanted valve prosthesis.

The present finding that there is a large inter-individual variation in EDACS is supported by observations in the past [21]. In the perspective of TAVI-induced LBBB, this variability of the membranous septum provides valuable insights into the pathophysiology and its predictors. Together with the small inter- and intra-observer variability (<1 mm), the measurement of EDACS enables accurate pre-procedural stratification of patients with low and high risk for developing P-LBBB.

The present data seem in line with previous studies [18,19] that showed an inverse relationship between membranous septum (MS) length and the risk of total AV block during TAVI procedures and permanent pacemaker implantation. Additionally, the reported range of MS lengths and the odds ratios related to MS length are comparable to the currently observed values (32–35% change) [18,19]. These similarities are even more notable because Hamdan et al. investigated self-expandable valves, and Maeno et al. investigated balloon-expandable valves [18,19], while the present study investigated both types of valves.

The predictive value of the pre-procedurally determined EDACS is further surprisingly good because it has been reported previously that several procedural and patient-related factors such as the implantation depth, a low ratio of annulus/balloon or annulus/prosthesis, self-expandable valves, aortic valve calcifications, increased QRS duration, pre-existent right bundle branch block (RBBB), diabetes and peripheral vascular disease also are predictive of the development of TAVI-induced LBBB, as reviewed in [13,14]. Our logistic regression analysis shows that prosthesis size, implantation depth and EDACS-ID do indeed contribute, to some extent, to the development of LBBB, but the effect of the EDACS seems to be considerably stronger.

### 4.2. Importance of Predicting P-LBBB

Being able to predict the development of P-LBBB during a TAVI procedure appears relevant because several studies showed poorer recovery of the LV ejection fraction, a higher rate of hospitalization and higher PPM implant rates after TAVI in patients who developed LBBB vs. those who did not [9,10,12]. Furthermore, several studies, including a quite recent one, showed an increased mortality in TAVI patients who developed LBBB as compared to those who kept a normal conduction [4,22]. This finding has been contradicted by a few other studies, which did not find such significant difference, but several explanations can be given for this discrepancy, such as the inclusion of patients in the LBBB group who received a permanent pacemaker [23,24], protecting them against bradyarrhythmic death.

Therefore, there is sufficient reason to avoid the development of LBBB during TAVI as much as possible.

### 4.3. Differences with Previous Studies

There were several findings in the present study that seem to differ from previous studies. First of all, the percentage of P-LBBB patients was similar in patients receiving balloon- and self-expandable valves (23 vs. 26%), whereas many previously published studies showed a lower percentage of P-LBBB in patients receiving a self-expandable valve [4,9,10,11,24].

The similar percentage of P-LBBB between these valve types occurred even in the presence of a deeper implantation of the self-expandable valves. This is also at odds with previous studies reporting that deeper valve implantation increases the chance of LBBB [3,5,25,26].

Several reasons can be postulated for the discrepancies between the findings in the present study and previous ones. The first papers on implantation depth originate from the starting period of TAVI. It has been noted that implanting experience is an important determinant of TAVI-induced LBBB, especially in the case of using self-expanding valves [26]. The present study contains data from patients implanted during recent years (as of 2011), largely after the aforementioned “learning curve” paper [26]. Moreover, the newer Sapien 3 valve seems to create more conduction disturbances than the older ones (~20% rather than ~10%) [27]. Therefore, it appears that with the current implantation experience and tools, the role of the valve type in causing TAVI-induced LBBB may have become smaller.

### 4.4. Clinical Implications

The major advantage of EDACS as a predictor for P-LBBB is the fact it can be determined preoperatively and can be taken into account in the preoperative planning of a patient with aortic valve stenosis. Given the abovementioned adverse effects of P-LBBB, the estimated risk for developing P-LBBB during TAVI may be worth implementing in deciding whether a patient would better undergo transcatheter or surgical aortic valve replacement. A large recent study showed that the chance of developing P-LBBB during surgical valve replacement is <2% [28]. This is as low as the risk during TAVI in patients with an EDACS of >10 mm, but more than 25 times lower than in patients with an EDACS of ≤3 mm. Therefore, especially for the latter group of patients, surgical replacement might be considered a preferred approach.

### 4.5. Limitations

The following limitations are to be recognized. This is a non-randomized study performed in two centers. The patients included were consecutive patients, but due to study requirements (CT scan and multiple ECGs), a large number of patients had to be excluded from the original 694 patients. CT scan acquisition was performed on different scanners in both centers; this, however, provides a more realistic real-life situation for hospitals with different CT scanners. Data were analyzed by an expert resident on conduction abnormalities and TAVI imaging. Numbers in the self-expandable valve group are relatively small.

## 5. Conclusions

A short EDACS is a positive predictor for the development of P-LBBB during a TAVI procedure, irrespective of the prosthesis type. Given the adverse effect of P-LBBB, EDACS is an important aid in pre-procedural decision making for aortic valve replacement procedures such as TAVI.

### Impact on Daily Practice

P-LBBB has previously been proven to worsen clinical outcomes of TAVI procedures. The present results imply that EDACS, determined preoperatively on the mandatory CT scan, may be a valuable additional factor to weigh the risks of transcatheter and surgical aortic valve replacement.

## Figures and Tables

**Table 1:** Baseline and procedural characteristics between the conduction groups.

Baseline Characteristics	Total (*n* = 269)	P-LBBB (*n* = 64)	Non-P-LBBB (*n* = 205)	*p*-Value
Age (years)	82 (78–84)	81 (77–84)	82 (78–85)	0.94
Female (*n*)	148 (55)	33 (52)	115 (56)	0.53
BMI (kg/m^2^)	26 (24–29)	26 (24–29)	26 (23–28)	0.29
Logistic EuroScore (%)	13 (9–20)	14 (10–23)	12 (8–19)	0.27
AF (*n*)	87 (32)	20 (31)	67 (33)	0.77
AMI (*n*)	45 (17)	9 (14)	36 (18)	0.56
PCI (*n*)	79 (29)	24 (38)	55 (27)	0.09
CABG (*n*)	60 (22)	11 (17)	49 (24)	0.29
CVA (*n*)	39 (15)	8 (13)	31 (15)	0.62
TIA (*n*)	9 (3)	2 (3)	7 (3)	0.93
PAD (*n*)	60 (22)	11 (17)	49 (24)	0.29
Diabetes (*n*)	81 (30)	22 (34)	59 (29)	0.40
Creatinin (umol/L)	93 (77–115)	94 (79–130)	91 (75–114)	0.12
COPD (*n*)	61 (23)	15 (23)	46 (22)	0.82
Hypertension (*n*)	221 (82)	49 (77)	172 (84)	0.18
Hypercholesterolemia (*n*)	197 (73)	41 (64)	156 (76)	0.07
ECG				
PRpre (ms)	174 (153,194)	183 (151,204)	174 (155,190)	0.24
QRSpre (ms)	94 (87,102)	98 (89,104)	94 (86,102)	<0.05
Echocardiogram				
LVEF (%)	60 (54,60)	60 (56,60)	60 (53,60)	0.77
Procedural factors				
*Access Route*				0.89
TF (*n* = 183)	183 (68)	43 (67)	140 (68)	
TA (*n* = 73)	73 (27)	19 (30)	54 (26)	
TAO (*n* = 13)	13 (5)	2 (3)	11 (5)	
*Prosthesis type*				0.60
Balloon-expandable (*n* = 200) *	200	46 (23)	154 (77)	
Self-expandable (*n* = 69) *	69	18 (26)	51 (74)	
*Prosthesis size*				<0.05
20 (mm)	1 (0)	0 (0)	1 (1)	
23 (mm)	79 (29)	13 (20)	66 (32)	
25 (mm)	11 (4)	2 (3)	9 (4)	
26 (mm)	107 (40)	26 (41)	81 (40)	
27 (mm)	6 (2)	2 (3)	4 (2)	
29 (mm)	53 (20)	16 (25)	37 (18)	
31 (mm)	12 (5)	5 (8)	7 (3)	
*Balloon aortic valvuloplasty (n = 244)*	244 (91)	58 (91)	186 (91)	0.98

Categorical variables are presented as numbers and proportions. Normal and skewed continuous variables are presented as means with standard deviation and medians with interquartile range (IQR). AF, atrial fibrillation; AMI, acute myocardial infarction; BMI, body mass index; CABG, coronary artery bypass grafting; COPD, chronic obstructive pulmonary disease; CVA, cerebrovascular accident; LVEF, left ventricular ejection fraction; mm, millimeter; ms, milliseconds; PAD, peripheral arterial disease; PCI, percutaneous coronary intervention; P-LBBB, persistent left bundle branch block; non-P-LBBB, no persistent left bundle branch block; TA, transapical; TAO, transaortic; TF, transfemoral; TIA, transient ischemic attack. * percentages are calculated per row.

**Table 2:** Measurements according to conduction groups in all patients.

	P-LBBB (*n* = 64)	T-LBBB (*n* = 49)	nQRS (*n* = 156)	*p*-Value (All Groups)
EDACS (mm)	4.6 (2.2,7.1)	8.2 (5.1,10.6)	7.8 (5.8,10.2)	<0.05
Implantation depth (mm)	6.9 (5.2,10.8)	7.0 (4.8,9.2)	6.0 (4.4,8.4)	<0.05
EDACS-ID (mm)	−2.6 (−6.9,0.3)	0.3 (−2.5,4.6)	1.4 (−1.3,4.8)	<0.05

EDACS, effective distance between aortic valve and conduction system; EDACS-ID, effective distance between aortic valve and conduction system–implantation depth; mm, millimeter; nQRS, narrow QRS complex; P-LBBB, persistent left bundle branch block; T-LBBB, temporary left bundle branch block. Skewed continuous variables are presented as medians with interquartile range (IQR).

**Table 3:** Measurements according to prosthesis type in all patients.

	All Patients	Balloon-Expandable Valves (*n* = 200)	Self-Expandable Valves (*n* = 69)	*p*-Value
EDACS (mm)	7.3 (4.9,9.7)	7.3 (4.9,9.8)	7.2 (4.7,9.1)	0.67
Implantation depth (mm)	6.3 (4.6,9.2)	5.8 (4.4,7.6)	9.6 (6.6,12.7)	<0.05
EDACS-ID (mm)	0.5 (−2.8,4.3)	1.1 (−1.8,4.6)	−2.3 (−5.4,1.4)	<0.05

EDACS, effective distance between aortic valve and conduction system; EDACS-ID, effective distance between aortic valve and conduction system–implantation depth mm, millimeter. Skewed continuous variables are presented as medians with interquartile range (IQR).

**Table 4:** Univariate and multivariate regression analysis for TAVI-induced P-LBBB.

	Univariate Analysis			Multivariate Analysis		
	OR	95% CI	*p*-Value	OR	95% CI	*p*-Value
Diabetes	1.30	0.71–2.36	0.40			
PAD	0.67	0.33–1.39	0.29			
Self-expandable valves	1.18	0.63–2.22	0.60			
Prosthesis size (mm)	1.16	1.03–1.30	<0.05	1.12	0.96–1.31	0.15
EDACS (mm)	0.68	0.60–0.77	<0.05	0.68	0.60–0.77	<0.05
Implantation depth (mm)	1.11	1.03–1.19	<0.05	1.08	0.99–1.18	0.09

PAD, peripheral arterial disease; EDACS, effective distance between aortic valve and conduction system, mm, millimeter.

## Data Availability

Not applicable.
